# ASO Author Reflections: Resected Intraductal Papillary Mucinous Neoplasm-Derived Pancreatic Cancer: Early Recurrence and Patient-Tailored Management

**DOI:** 10.1245/s10434-024-16810-8

**Published:** 2025-01-24

**Authors:** Joseph R. Habib, Ingmar F. Rompen, Camila Hidalgo Salinas, Vincent P. Groot, Ammar A. Javed, Lois A. Daamen

**Affiliations:** 1 Department of Surgery, New York University Langone Health, New York, NY USA; 2 Department of Surgery, Regional Academic Cancer Center Utrecht, UMC Utrecht Cancer Center, St. Antonius Hospital Nieuwegein, Utrecht, The Netherlands; 3 Department of Surgery, Amsterdam UMC, University of Amsterdam, Amsterdam, The Netherlands; 4 Cancer Center Amsterdam, Amsterdam, The Netherlands; 5 Division of Imaging and Oncology, University Medical Center Utrecht, Utrecht, The Netherlands

## Past

The available literature on the natural progression, management, and outcomes of pancreatic cancer originating from intraductal papillary mucinous neoplasm (IPMN) remains limited. Consequently, after resection, these neoplasms have traditionally been treated and monitored according to the more comprehensively studied pancreatic intraepithelial neoplasm (PanIN)-derived pancreatic cancer.

## Present

Recent evidence has demonstrated that almost half of patients with IPMN-derived pancreatic cancer succumb to local or distant disease recurrence within 5 years after pancreatic resection.^1^ This lower propensity for recurrence in IPMN-derived pancreatic cancer as compared with PanIN-derived pancreatic cancer supports a more indolent biology, making it reasonable to suspect that optimal postoperative treatment and surveillance may require different strategies.^2^ The present study establishes high-, intermediate-, and low-risk cohorts of patients for early recurrence (within 11 months of surgery for IPMN-derived pancreatic cancer), which is associated with poorer survival after recurrence than late or no recurrence.^3^ The present study also revealed that patients with resected IPMN-derived pancreatic cancer can be risk-stratified based on serum carbohydrate antigen 19-9 (CA19-9) and American Joint Commission on Cancer (AJCC) N-stage.^3^ The presence of elevated CA19-9 and nodal disease has already been implicated in identifying patients who may have an associated benefit from adjuvant chemotherapy.^4^ The results of the present study support this conclusion. Moreover, these risk-stratified cohorts exhibited differing rates of early recurrence ranging from as low as 1% (low-risk cohort) to as high as 32% (high-risk cohort).^3^ The varying likelihoods of early recurrence could be helpful to inform risk-stratified surveillance strategies. It is especially important to diagnose and treat disease recurrence in a timely manner, given the associated improved survival in patients who received treatment for recurrence.^1^

## Future

Emerging evidence suggests that IPMN- and PanIN-derived pancreatic cancer are clinically and biologically distinct, emphasizing the need for further evidence into tailored management strategies for these patients.^2,5^ In particular, prospective evidence is urgently needed to identify and validate optimal treatment strategies for resected IPMN-derived pancreatic cancer.
